# Extracellular Vesicles Analysis in the COVID-19 Era: Insights on Serum Inactivation Protocols towards Downstream Isolation and Analysis

**DOI:** 10.3390/cells10030544

**Published:** 2021-03-04

**Authors:** Roberto Frigerio, Angelo Musicò, Marco Brucale, Andrea Ridolfi, Silvia Galbiati, Riccardo Vago, Greta Bergamaschi, Anna Maria Ferretti, Marcella Chiari, Francesco Valle, Alessandro Gori, Marina Cretich

**Affiliations:** 1 Istituto di Scienze e Tecnologie Chimiche “Giulio Natta” (SCITEC), Consiglio Nazionale delle Ricerche, Via Alfonso Corti 12, 20133 Milano, Italy; roberto.frigerio94@gmail.com (R.F.); angelo.musico94@gmail.com (A.M.); greta.bergamaschi@cnr.it (G.B.); anna.ferretti@cnr.it (A.M.F.); marcella.chiari@cnr.it (M.C.); 2 Istituto per lo Studio dei Materiali Nanostrutturati (ISMN), Consiglio Nazionale delle Ricerche, Via P. Gobetti 101, 40129 Bologna, Italy; marco.brucale@cnr.it (M.B.); andrea.ridolfi@ismn.cnr.it (A.R.); francesco.valle@cnr.it (F.V.); 3 Consorzio Interuniversitario per lo Sviluppo dei Sistemi a Grande Interfase (CSGI), Via della Lastruccia 3, Sesto Fiorentino, 50019 Firenze, Italy; 4 IRCCS San Raffaele Scientific Institute, Via Olgettina 60, 20132 Milano, Italy; galbiati.silvia@hsr.it (S.G.); vago.riccardo@hsr.it (R.V.)

**Keywords:** extracellular vesicles, COVID-19, SARS-CoV-2, virus inactivation, biosafety, pre-analytical protocols, human serum, serum specimen

## Abstract

Since the outbreak of the COVID-19 crisis, the handling of biological samples from confirmed or suspected SARS-CoV-2-positive individuals demanded the use of inactivation protocols to ensure laboratory operators’ safety. While not standardized, these practices can be roughly divided into two categories, namely heat inactivation and solvent-detergent treatments. These routine procedures should also apply to samples intended for Extracellular Vesicles (EVs) analysis. Assessing the impact of virus-inactivating pre-treatments is therefore of pivotal importance, given the well-known variability introduced by different pre-analytical steps on downstream EVs isolation and analysis. Arguably, shared guidelines on inactivation protocols tailored to best address EVs-specific requirements will be needed among the analytical community, yet deep investigations in this direction have not yet been reported. We here provide insights into SARS-CoV-2 inactivation practices to be adopted prior to serum EVs analysis by comparing solvent/detergent treatment vs. heat inactivation. Our analysis entails the evaluation of EVs recovery and purity along with biochemical, biophysical and biomolecular profiling by means of a set of complementary analytical techniques: Nanoparticle Tracking Analysis, Western Blotting, Atomic Force Microscopy, miRNA content (digital droplet PCR) and tetraspanin assessment by microarrays. Our data suggest an increase in ultracentrifugation (UC) recovery following heat treatment; however, it is accompanied by a marked enrichment in EVs-associated contaminants. On the other hand, solvent/detergent treatment is promising for small EVs (<150 nm range), yet a depletion of larger vesicular entities was detected. This work represents a first step towards the identification of optimal serum inactivation protocols targeted to EVs analysis.

## 1. Introduction 

The COVID-19 pandemic has forced researchers to deal with clinical specimens from confirmed or suspected SARS-CoV-2-positive cases. Current biocontainment guidelines to address lab operators’ exposure risk are adopted according to international standards and constantly updated (https://www.cdc.gov/coronavirus/2019-nCoV/lab/lab-biosafety-guidelines.html (accessed on 8 Februaty 2021)). In this regard, the minimum biosafety level to handle suspected SARS-CoV-2 specimens during non-propagative procedures is Biosafety Level-2 (BSL-2), provided that the samples have been biologically inactivated to abolish or mostly suppress virus infectivity. These precautionary actions are taken even if viral-inactivating methods are more relevant for blood-borne viruses such as HBV, HCV, HIV and arboviruses than for respiratory viruses. Common inactivation protocols for SARS-CoV-2 are inherited from previous studies on enveloped viruses validated during the past MERS or SARS outbreaks; those preceding molecular diagnostics (involving RNA extraction) are usually based on chemical treatments with detergents and chaotropic agents [1,2]. Previous experience of the serology of coronaviruses also suggested treatments with a solvent-detergent combination (e.g., Triton X100/Tween 80 and tri(n-butyl) phosphate), as currently adopted for serum/plasma standards by the Medicine & Healthcare products Regulatory Agency [3]. Heat treatment is another routine inactivation method, especially for serum/plasma. On this matter, while data are still debated [1,4,5], serum heat inactivation at 56 °C for 30 min is emerging as a common practice. 

In this scenario, arguably, it is anticipated that assessing the impact of different serum inactivation protocols on downstream Extracellular Vesicles (EVs) isolation and analysis will be of primary relevance to the EVs field, while, to the best of our knowledge, no investigation has been reported so far in this direction. Human blood is an important source of accessible EVs originating from various body cell types with significant interest as potential diagnostic biomarkers, yet their isolation represents a considerable challenge. EVs from biological samples are indeed tremendously complex analytes, and the well-known influence of pre-analytical practices on downstream EVs use has been driving the need for standardization and rigor criteria largely before the COVID crisis, as highlighted by the International Society for Extracellular Vesicles (ISEV) community [6,7].

**Scheme 1:**
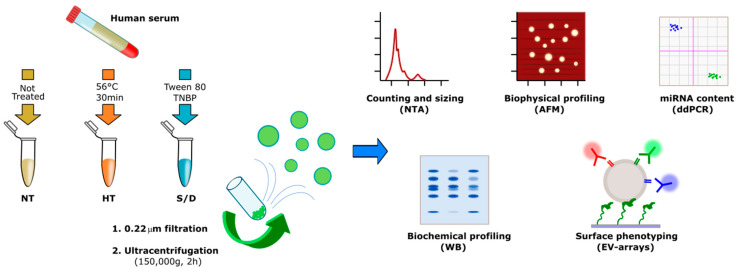
Workflow describing the sample treatments and Extracellular Vesicle (EV) isolation and characterization. The number of samples analyzed by each technique is reported in Table S1 in the Supplementary Material.

Herein, we report on the influence of two common COVID-19 serum-inactivation protocols on EVs recovery, purity, biophysical, biochemical and biomolecular traits. Specifically, on unbiased premises, we focused our investigation on EVs isolated by ultracentrifugation (UC) from untreated (NT), heat-treated (HT) and solvent/detergent (S/D)-treated healthy sera. Samples were analyzed by a set of complementary techniques namely Nanoparticle Tracking Analysis (NTA), Western Blotting (WB), Atomic Force Microscopy (AFM), miRNA 16-5p and miRNA 21-5p quantification by droplet digital PCR (ddPCR) and microarrays for tetraspanin assessment. A flow chart of the experimental strategy is shown in Scheme 1. Our data showed that serum heat inactivation provided the highest recovery after UC, yet inclusive of contaminants enrichment. Solvent/detergent treatment led to no remarkable effects when small EVs (< 150 nm range) were considered, providing the best EVs purity among the three groups. Far from being conclusive, our work aims to provide preliminary insights and awareness among the EVs community on viruses inactivation practices to be adopted prior to serum EVs analysis. 

## 2. Experimental Section

### 2.1. Samples

Sixteen serum samples purchased from Cerba Expert (Saint Ouen L’Aumone, France) and collected from healthy subjects before December 2018 were used. Blood samples were collected in BD VACUTAINER (clot activator tube). Serum samples were separated after centrifugation within two hours from blood collection at 1900× *g* for 10 min at 4 °C. Specimens were stored at −20 °C until use. 

### 2.2. Ultracentrifugation

A total of 750 µL of serum were diluted 1:1 with PBS, filtered with 0.22 mm filters (Merck Millipore, Darmstadt, Germany) and centrifuged in an Optima™ TLX Preparative Ultracentrifuge, Beckman Coulter^TM^ at 150,000× *g* for 120 min at 4 °C with a *TLA-55* Rotor (Beckman Coulter^TM^, Brea, CA, USA) to pellet EVs. After supernatant was carefully removed, EV-containing pellets were stored at −80 °C until use. 

### 2.3. Nanoparticle Tracking Analysis 

Nanoparticle tracking analysis (NTA) was performed according to the manufacturer’s instructions using a NanoSight NS300 system (Malvern Technologies, Malvern, UK) configured with a 532  nm laser. All samples were diluted in filtered PBS to a final volume of 1 mL. Ideal measurement concentrations were found by pre-testing the ideal particle per frame value (20–100 particles/frame). The following settings were adjusted according to the manufacturer’s software manual. A syringe pump with constant flow injection was used and three videos of 60 s were captured and analyzed with NTA software version 3.2. From each video, the mean, mode, and median EVs size was used to calculate samples concentration, expressed in nanoparticles/mL.

### 2.4. Protein Quantification

We performed Bradford Assay to quantify protein concentration on our samples. The samples were added in a Bradford solution (BioRad Protein Assay 500-0006, Hercules, CA, USA) 1:5 diluted in water and analyzed by a spectrophotometer (Labsystem, Multiskan Ascent, Missouri, TX, USA) at a wavelength of 595 nm. Furthermore, we analyzed standard protein solutions to build a calibration line to discover the right protein concentration of our samples. 

### 2.5. SDS-PAGE and Western Blot Analysis

Treated EVs were added to Laemmli buffer and boiled for 5 min at 95  °C. Specifically, 10  µg of EVs were prepared in non-reducing conditions for tetraspanins detection, while 10  µg were used for soluble protein detection. Proteins were separated by SDS-PAGE (4–20%, Mini-Protean TGX Precast protein gel, Bio-Rad) and transferred onto a nitrocellulose membrane (BioRad, Trans-Blot Turbo). Nonspecific sites were saturated with a blocking solution for 1 h (EveryBlot Blocking Buffer, BioRad). Membranes were incubated overnight at 4  °C with anti-CD9 (1:1000, BD Pharmingen), anti-CD63 (1:1000; BD Pharmingen, San Jose, CA, USA), anti-Alix (1:1000, Santa Cruz, CA, USA), anti-TSG101 (1:1000, Novus Bio, Centennial, CO, USA) and anti-Apo1 (1:1000, Santa Cruz). After washing with T-TBS, membranes were incubated with the horseradish peroxidase-conjugated (Jackson ImmunoResearch, Tucker, GA, USA) secondary antibodies diluted 1:3000 for 1 h. After washing, the signal was detected using Bio-Rad Clarity Western ECL Substrate (Bio-Rad) and imaged using a Chemidoc XRS+ (BioRad).

### 2.6. AFM Sample Preparation, Imaging and Morphometry

Borosilicate glass coverslips (Menzel Gläser GmbH, Braunshweig, Germany) were first incubated for 1 h in 2:1 H_2_SO_4_:H_2_O_2_ (30% *v/v*) solution, then rinsed with ultrapure water, subjected to 3 × 30 min successive sonication cycles in acetone, isopropanol and ultrapure water, and finally dried under gentle nitrogen flow. Glass slides were then exposed for 5 min to air plasma and functionalized with (3-Aminopropyl)triethoxysilane (APTES) in vapor phase for 2 h. Resuspended EVs solutions were diluted 1:100 with ultrapure water; 5 μL aliquots of the diluted solutions were then left to adsorb on APTES-functionalized slides for 30 min. AFM imaging was performed in PeakForce mode on a Multimode8 AFM microscope equipped with a type JV scanner and a sealed fluid cell (Bruker, Billerica, MA, USA). Image analysis was performed as described elsewhere [8].

### 2.7. EV Array

Silicon slides (SVM, Sunnyvail, CA, USA) were coated by MCP6 polymers (Lucidant Polymers, Sunnyvale, CA, USA) and spotted with BP membrane binding peptide (RPPGFSPRKG) synthesized as described in [9]. Printed slides were placed in a humid chamber overnight at room temperature. EV samples were incubated for 2 h at a particle concentration of 10 [10] particles/mL. Slides were then washed with washing buffer and incubated with anti-CD9-Biotin, anti-CD63-Biotin and anti-CD81-Biotin antibodies (Ancell) 0.1 mg/mL for 1 h and with Streptavidin-Cy3 (Jackson ImmunoResearch) 0.1 mg/mL for 1 h. Finally, slides were washed and dried, and the analyses were performed by TECAN power scanner 50% laser intensity and 500% gain.

### 2.8. miRNA Isolation and Retrotrascription

miRNAs were isolated from ultracentrifuged EVs resuspended in 25 µL of phosphate-buffered saline (PBS) using the Maxwell^®^ RSC miRNA Plasma and Serum Kit (AS1680, Promega, Madison, WI, USA) following the manufacturer’s instruction. The RNA was eluted in 35 µL of nuclease-free water.

cDNA was obtained using the TaqMan^®^ MicroRNA Reverse Transcription kit (ThermoFisher, Waltham, MA, USA) combined with TaqMan MicroRNA Assays (ThermoFisher). In particular, we used 5 µL of eluted RNA and 3 µL of primers specific for human miR-16 (assay ID 000391) and miR-21 (ID 000397). The reaction was performed with an initial incubation at 16 °C for 30 min and the following step at 42 °C for 30 min, finally; in order to terminate the RT step, a final incubation at 85 °C for 5 min was completed.

### 2.9. ddPCR Reagents and Cycling Conditions

The miR-16-5p and miR-21-5p expression levels were performed by droplet digital PCR (ddPCR) using the QX100 ddPCR platform (Bio-Rad, Hercules, CA, USA). The QX100 droplet generator was used to generate an emulsion of about 20,000 droplets. The volume of the PCR mix was 20 µL including 10 µL of ddPCR™ Supermix for Probes (No dUTP, Hercules, CA, USA), 1 µL of probe (miR-16 or miR-21) and 5 µL of cDNA template. The droplet emulsion was thermally cycled on C1000 Touch Thermal Cycler (Bio-Rad) instrument. Cycling conditions were 95 °C for 5 min, followed by 40 cycles of amplification (94 °C for 30 s and 55 °C for 1 min), ending with 98 °C for 10 min, according to the manufacturer’s protocol. The concentration of the target was calculated automatically by the QuantaSoft™ software version 1.7.4 (Bio-Rad).

### 2.10. EV-TRACK

We have submitted all relevant data of our experiments to the EV-TRACK knowledgebase (EV-TRACK ID: EV200180) (Van Deun J. et al. *EV-TRACK: transparent reporting and centralizing knowledge in extracellular vesicle research.* Nature methods. 2017;14(3):228–32).

## 3. Results and Discussion

### 3.1. Sample Preparation 

Sixteen pre-COVID serum samples from healthy donors were divided in three aliquots (750 µL each). For each serum sample, one aliquot was left untreated (NT), one aliquot (HT) was set to mimic heat inactivation (56 °C for 30 min) and one aliquot (S/D) was treated with 10 mg/mL Tween 80 and 3 mg/mL tri(n-butyl) phosphate (TNBP). Hints on the compatibility of such treatments with EV integrity were previously reported in studies on the stability of vesicles upon different temperatures [10] and non-ionic detergents [11]. The resulting 48 samples were subjected to standard ultracentrifugation (UC) at 150,000× *g* for 2 h. It is well documented that single-step EVs isolation procedures, including UC, are likely to lead to EVs co-isolation of contaminants such as protein aggregates, VLDLs, LDLs and chylomicrons [12,13], whereas a combination of sequential purification steps provides increased purity [14,15]. We therefore reasoned that the simple and routinely performed EVs isolation by UC could be particularly indicative in assessing the role of serum pre-treatment on the extent of co-isolated contaminants. 

### 3.2. Nanoparticle Tracking Analysis 

**Figure 1:**
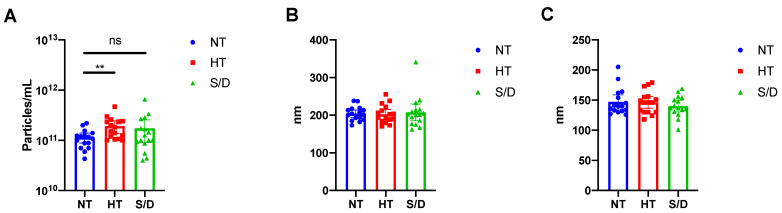
Nanoparticle Tracking Analysis (NTA) of EVs isolated by ultracentrifugation from untreated (NT), heat-treated (HT) and solvent/detergent (S/D) treated healthy sera. N = 16. (**A**): mean particle count with 95%CI. (**B**): mean particle size with 95% CI. (**C**): median particle size with 95% CI. Significance level: *p* < 0.05; ** = *p* < 0.01. Representative results of NTA analysis for NT, HT and S/D samples are reported in Figure S1, Supplementary Material.

Pellets from UC samples were resuspended in filtered PBS (50 μL), and particle number and sizing of the 48 samples were determined by Nanoparticle Tracking Analysis (NTA) as described in the Experimental Section. The resulting particle concentration (A), mean (B) and median (C) of particle diameter for the untreated (NT), heat-treated (HT) and solvent/detergent treated (S/D) samples are shown in Figure 1. 

NTA analysis revealed a significant (*p* < 0.01) increase in particle counting in the HT EVs compared to the NT sample, whereas no difference was detected at a statistically significant level for the S/D treated EVs (Figure 1A). However, the S/D samples showed a higher variability in the number of UC recovered particles (Figure 1A). As for particle mean and median size, no significant differences were found among the three sample sets. It is well known that given the presence of co-isolated lipoproteins, the quantification of EVs based on particle counting by NTA tends to overestimate EVs concentration [16]. Thus, we preliminarily hypothesized that the increased number of recovered particles that were observed after heat-treatment could be ascribed to the increased co-precipitation of lipoproteins and other protein aggregates triggered by heat-induced aggregation. 

### 3.3. Western Blotting 

Western blotting (WB) was used to confirm the presence of EVs transmembrane (CD9 and CD63) and luminal proteins (Alix and TSG101), as well as the presence of common co-isolated contaminants (Apolipoprotein A I, Apo AI) in a set of NT, HT and S/D samples. Prior to WB, protein concentration in the UC-recovered pellets was assessed by Bradford assay, showing a remarkably higher protein content in the HT sample (10.6 mg/mL vs. 3.8 mg/mL for both NT and S/D). The samples were then diluted and loaded on the gel at the same protein amount per lane (5 µg). 

**Figure 2:**
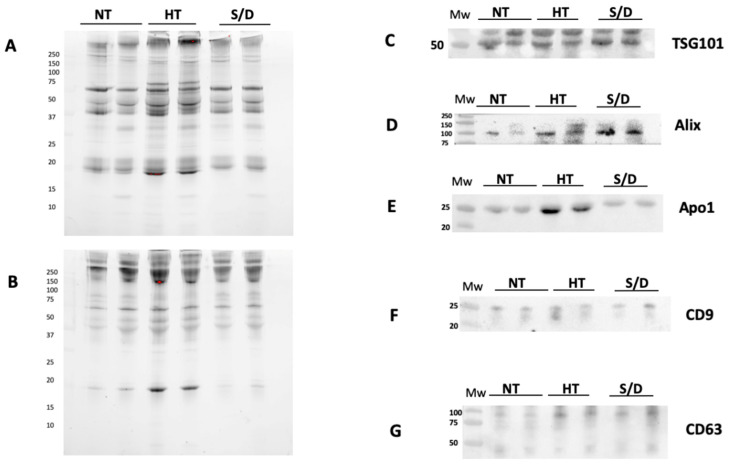
Western Blotting analysis of EVs isolated by ultracentrifugation (UC) from untreated (NT), heat-treated (HT) and solvent/detergent (S/D)-treated healthy sera. N = 12. The SDS PAGE of pellets was run in non-reducing conditions (**A**) and reducing conditions (**B**). Immunoblotting was performed for TSG101 (**C**); Alix (**D**); contaminant Apoliprotein AI (**E**); tetraspanin CD9 (**F**) and CD63 (**G**).

Figure 2 shows the results of the WB gels for two representative samples for each sample group, analyzed in non-reducing conditions (A), reducing conditions (B) and the corresponding immunoblotting for the assessment of TSG101 (C), Alix (D), CD9 (F), CD 63 (G). Overall, the presence of typical EVs markers was demonstrated for all three sample sets with similar isolation yields. However, a higher amount of co-isolated Apo AI is clearly detectable in the HT group (E). 

**Figure 3:**
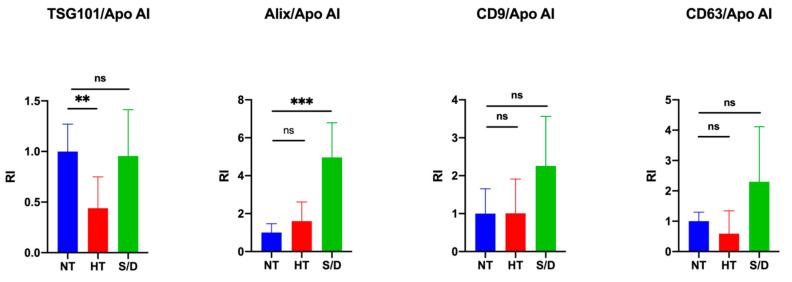
Quantification of blotted protein bands and ratio between EV luminal markers TSG101/Alix and EV surface markers CD9CD63 with contaminant lipoprotein Apo AI. Significance level: *p* < 0.05; ** = *p* < 0.01; *** = *p* < 0.001.

The quantification of intensity for each immune-blotted protein band was performed and averaged. We then calculated the ratio between EV-specific protein markers and the co-isolated Apo AI contaminant in order to estimate and compare the purity yield of isolated EVs after each inactivation treatment. Results are reported in Figure 3. An increase in co-isolated lipoproteins following heat treatment is clearly observable (*p* < 0.01) by comparing the ratio between the luminal marker TSG101 and Apo AI, whereas an increased purity in the S/D samples (*p* < 0.001) was observed considering the ratio between ALIX and Apo AI. On the other hand, no clear difference among the samples was detectable when the ratio between tetraspanins CD9/CD63 and Apo AI was considered. The selection of appropriate markers for such comparison, as a consequence, may prove extremely critical, posing multimarker selection as likely mandatory. Overall, an apparent reduction in lipoprotein contaminants was observed in the case of S/D treatment, even in comparison with untreated samples. This observation suggests a role of solvent/detergent in shielding those supramolecular interactions at the colloidal level among EVs and lipoproteins, which may contribute to contaminant co-isolation. 

### 3.4. Atomic Force Microscopy (AFM) 

**Figure 4:**
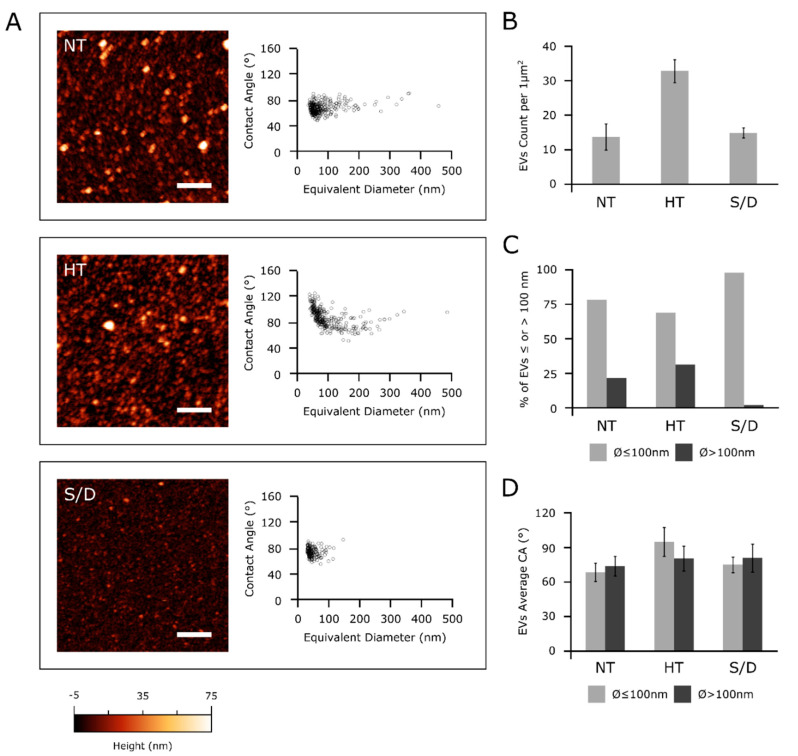
(**A**): left column—representative AFM micrographs of NT, HT and S/D samples. Scale bars are 1 μm. Right column—scatterplots of surface contact angle vs. diameter in solution of EVs measured via quantitative AFM morphometry as described elsewhere [8]. Each circle represents an individual EV. (**B**): surface density of globular objects in NT, HT and S/D samples deposited with the same protocol (see Experimental Section); (**C**) percentage of adsorbed EVs having diameters above or below 100 nm in their spherical conformation; (**D**) average surface/vesicle contact angle (representative of mechanical stiffness) of EVs with diameters above or below 100 nm.

Samples collected from the different serum inactivation protocols were analyzed via a high-throughput nanomechanical screening method described elsewhere [8]. Briefly, the vesicle/surface contact angle (CA) of individual EVs adsorbed on a substrate can be measured via AFM morphometry and used as a direct indication of their mechanical stiffness. The same procedure allows calculating the diameter of each observed EV prior to surface adsorption. Vesicular objects are characterized by a narrow distribution of CAs at all diameters, whereas non-vesicular contaminants show a wider dispersion of CAs [17], which can be used to infer their presence in a sample even when their globular morphology makes it difficult to discern them from EVs. Figure 4 summarizes the main differences revealed by AFM morphometric analysis across the panel of samples.

All examined EVs samples showed an abundant vesicular content (Figure 4A, left column); however, in accordance with the increased particle counting observed via NTA (Figure 1), the HT sample showed more than twice the amount of adsorbed globular objects (Figure 4B) with respect to NT and S/D samples deposited with the same procedure (see Experimental Section).

Figure 4A shows the CA vs. diameter plots of around 200-300 individual EVs for each sample. All three samples were found to contain a high proportion of globular objects with diameters in the 50–100 nm range and a decidedly smaller amount of objects with diameters between 100 and 500 nm. In particular, only 2% of the EVs in the S/D sample had a diameter above 100 nm (Figure 4C), with no individual S/D treated EV having a diameter >150 nm (Figure 4A). In contrast, a more substantial amount of EVs in both the other samples had diameters above 100 nm (respectively, 22% and 31% of the EVs measured in NT and HT samples, Figure 4C). 

Figure 4D shows the average CAs of EVs smaller and larger than 100 nm in the three samples. The NT CA/diameter scatterplot in Figure 4A does not show any significant CA discontinuity between the two ranges of diameters; accordingly, average CA values of smaller (68 ± 8°, N = 242) and larger (73 ± 8°, N = 67) EVs were similar in this sample (Figure 4D). The S/D sample shows very similar values (74 ± 7°, N = 171 for smaller and 79 ± 12°, N = 4 for larger EVs), suggesting that while the solvent/detergent treatment dramatically reduced the amount of larger EVs, it did not significantly impact the structural integrity of the remaining EVs, which continued to show the same mechanical characteristics of untreated ones. The same consideration can be made for larger EVs in HT samples (average CA = 79 ± 11°, N = 103). In contrast, globular objects with diameters below 100 nm had a significantly higher CA (94 ± 12°, N = 226) in HT, suggesting marked structural or compositional differences in this sub-population of objects with respect to other samples [12,17].

Taken together, these results suggest the possibility that NT samples might contain different types of vesicular objects sharing similar mechanical characteristics but having different average dimensions, with a diameter threshold of around 100 nm separating the main subpopulations. 

Although the absence of significant CA differences across all sizes in non-treated EVs makes this hypothesis only tentative, HT and S/D treatments seem to selectively act on only some of the putative subpopulations: S/D is able to deplete larger EVs, while HT enriched the solution with a population of objects with distinct mechanical properties. 

### 3.5. Microarray Analysis

**Figure 5:**
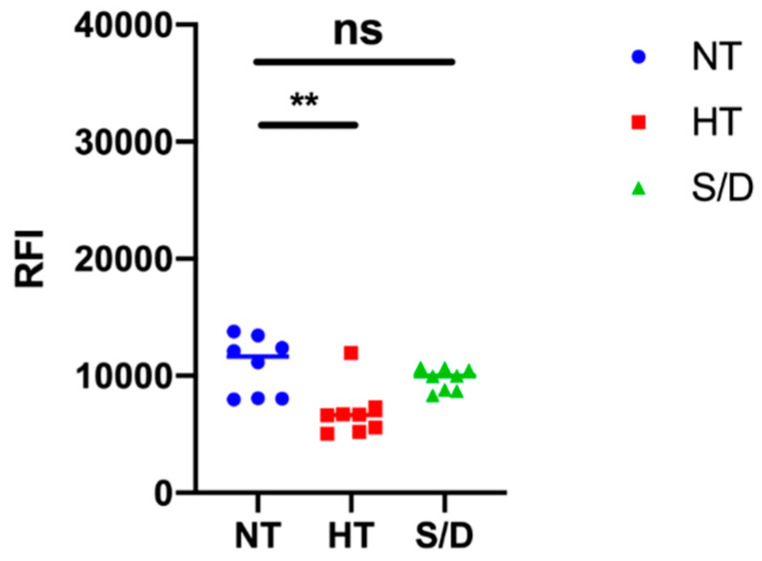
Results of immune-phenotyping by peptide microarrays of EVs isolated by ultracentrifugation from untreated (NT), heat treated (HT) and solvent/detergent (S/D) treated sera. EVs were captured by BP membrane binding peptide and fluorescently stained by a mixture of anti-CD9/CD63/CD81 antibodies. Significance level: *p* < 0.05; ** = *p* < 0.01.

EVs microarrays are high-throughput analytical platforms that are used to phenotype EVs. In this technique, antibodies [18] or peptide ligands [9] are used to capture EVs by their most common surface-associated proteins or by membrane sensing, followed by fluorescence immune-staining of membrane biomarkers. Here, the BP membrane binding peptide [9] that captures EVs by a general, membrane-mediated mechanism not involving surface antigens was immobilized on a silicon-based microarray platform for enhanced fluorescence detection [19,20]. EVs from the NT, HT and S/D groups were then incubated at 10^9^ particles/mL, and a cocktail of biotinylated anti-CD9/CD63/CD81 antibodies followed by Cy3-labeled streptavidin was used for detection. Figure 5 reports the resulting fluorescence intensities for each group of samples. Results show a decrease in immune-reactivity particularly pronounced in the HT-inactivated samples, which could suggest a lower content in tetraspanin-responsive particles; on the other hand, we cannot rule out a possible partial denaturation of EVs surfaces markers affecting immune-staining. Overall, sample treatment appears to not preclude the possibility of EVs immune-phenotyping, but the overall picture could prove tricky to unambiguous define. 

### 3.6. miR-16-5p and miR-21-5p ddPCR Analysis

Among the different RNA classes, microRNAs (miRNA) are abundantly harbored in many body fluids via encapsulation and/or association with EVs as well as transported by lipoproteins [21], which avoid nucleolytic degradation.

We selected two representative miRNAs, namely miR-16-5p and miR-21-5p, to compare if/how serum inactivation protocols could influence the miRNA levels.

**Figure 6:**
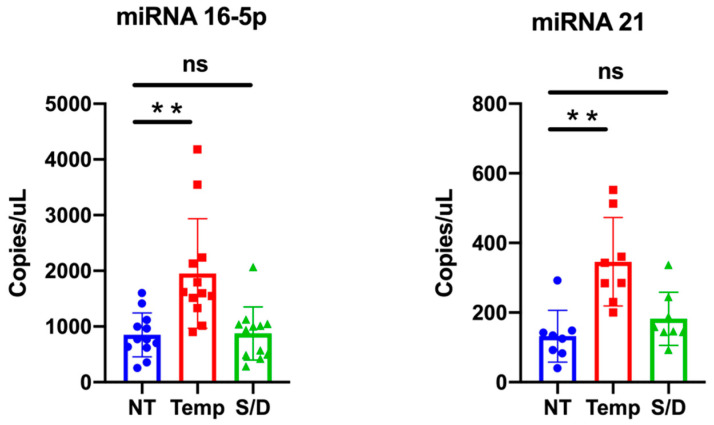
miR-16-5p and miR-21-5p expression levels in untreated (NT), heat treated (HT) and solvent/detergent (S/D)-treated healthy sera analyzed by droplet digital PCR. Significance level: *p* < 0.05; ** = *p* < 0.01.

MiR-16-5p is among the most abundant miRNA in EVs [22], while miR-21-5p is reported to be associated withs lipoproteins and could be involved in lipid metabolism [23]. We quantitatively detected miRNAs levels within NT, HT and S/D groups by ddPCR analysis; results are summarized in Figure 6. Data analysis highlights a clear trend for both miR-16-5p and miR-21-5p, with an increased amount detected within the HT samples. This observation is consistent with the apparent higher EVs isolation yield for the HT samples suggested by protein quantification and NTA. On the other hand, given the reported data on EVs purity (see WB Section) that instead suggested that HT samples contained higher levels of co-isolated contaminants, a more comprehensive interpretation should take into account the reported association of RNAs also to RNA-binding proteins (RBPs) and, especially for miRNA 21-5p, to high- and low-density lipoproteins [21,24].

## 4. Conclusions

Our study was aimed at introducing preliminary insights on the role of different SARS-CoV2 inactivation protocols prior to serum EVs isolation and analysis. To achieve this aim, we focused on collective biochemical and biophysical properties in a set of serum samples from healthy controls reflecting the well-known heterogeneity and biological variability of EVs in blood. Exacerbated by the current pandemic scenario, this topic will arguably gain broad relevance to the analytical community. Clinical sample preparation is indeed known to have a profound impact on the isolation of EVs and related contaminants [6]. A full awareness of the effect of any additional sample pre-analytical treatment should be consequently raised among EVs users, particularly due to the fact that an interlaboratory consensus on protocols for SARS-CoV2 serum inactivation protocols is far from being reached. In this sense, laboratories that use bio-banked samples collected by clinicians may be particularly affected. 

Far from being conclusive, our data suggest that the use of solvent/detergent addition could be seen as a preferable virus-deactivating method, taking EV’s purity obtained after a single UC step into account and as far as small EVs isolation and analysis are concerned. Non-ionic detergents are indeed relatively mild and usually non-denaturing [16], but they are known to break lipid–lipid and lipid–protein interactions. This could account for the apparent higher purity of EVs obtained after S/D treatment (see WB analysis), which contrasts with the higher content of lipoparticle contaminants detected following heat treatment. In this sense, their use of upfront UC cycles could be worth further investigating. On the other hand, solvent/detergent treatment led to the depletion of vesicular particles of larger size (>150 nm), and their use should be cautiously pondered if this EVs subpopulation represents the target of analysis. In contrast, heat-based protocols provided higher recovery, and the enrichment in EVs contaminants could be counterbalanced by the introduction of a subsequent step of purification. Overall, the virus-inactivation procedure should be tailored considering the downstream analysis to be undertaken, and further work will be needed in this direction to identify the best possible practices. 

## Supplementary Materials

The following are available online at https://www.mdpi.com/2073-4409/10/3/544/s1. Supplementary Table S1: number of serum samples for each group (NT, S/D, HT) analyzed by each technique; Supplementary Figure S1: representative NTA analysis graph for the non treated (NT), Heat Treated (HT) and solvent/detergent (S/D) treated sample.
