# Supplementary material for: Extracellular Vesicles Analysis in the COVID-19 Era: Insights on Serum Inactivation Protocols towards Downstream Isolation and Analysis
Source: Cells. 2021 Mar 4;10(3):544. doi: 10.3390/cells10030544 (PMC8001372; doi:10.3390/cells10030544)
Supplement: Supplementary file 1 [file cells-10-00544-s001.pdf]

Technical Note

Extracellular Vesicles Analysis in the COVID-19 Era: Insights on Serum Inactivation Protocols Towards Downstream Isolation and Analysis

Roberto Frigerio [1,†], Angelo Musicò [1,†], Marco Brucale [2,3], Andrea Ridolfi [2,3], Silvia Galbiati [4], Riccardo Vago [4],
Greta Bergamaschi [1], Anna Maria Ferretti [1], Marcella Chiari [1], Francesco Valle [2,3], Alessandro Gori [1,*,‡] and Marina Cretich [1,*,‡]

[1] Istituto di Scienze e Tecnologie Chimiche "Giulio Natta" (SCITEC), Consiglio Nazionale delle Ricerche,
Via Alfonso Corti 12, 20133 Milano, Italy; roberto.frigerio94@gmail.com (R.F.); angelo.musico94@gmail.com (A.M.); greta.bergamaschi@cnr.it (G.B.); anna.ferretti@cnr.it (A.F.); marcella.chiari@cnr.it (M.C.)
[2] Istituto per lo Studio dei Materiali Nanostrutturati (ISMN), Consiglio Nazionale delle Ricerche, Via P. Gobetti 101, 40129 Bologna, Italy; marco.brucale@cnr.it (M.B.); andrea.ridolfi@ismn.cnr.it (A.R.);
francesco.valle@cnr.it (F.V.)
[3] Consorzio Interuniversitario per lo Sviluppo dei Sistemi a Grande Interfase (CSGI), Via della Lastruccia 3, 50019 Sesto Fiorentino, Firenze, Italy
[4] IRCCS San Raffaele Scientific Institute, Via Olgettina 60, 20132 Milano, Italy; galbiati.silvia@hsr.it (S.G.); vago.riccardo@hsr.it (R.V.)
* Correspondence: alessandro.gori@cnr.it (A.G.); marina.cretich@cnr.it (M.C.)
† these authors equally contributed.
‡ these authors equally contributed.

Table S1: number of serum samples for each group (NT, S/D, HT) analyzed by each technique

Technique	Number of analyzed samples per group
Nanoparticle Tracking Analysis	16
Western Blotting	12
Atomic Force Microscopy	1
Microarrays	8
ddPCR Analysis	8

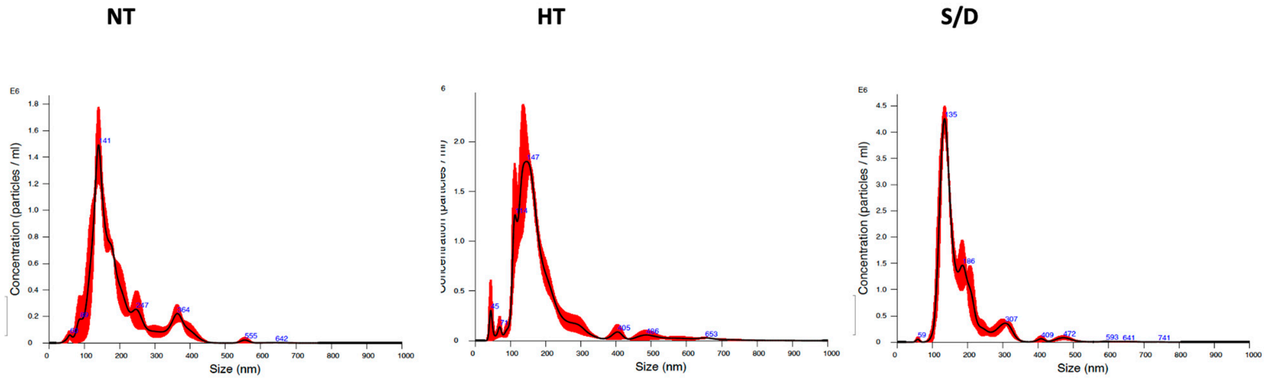

Figure S1: representative NTA analysis graph for the non treated (NT), Heat Treated (HT) and solvent/detergent (S/D) treated sample
